# Pre-cooling of boar semen before transport in a hot environment enhances biosafety

**DOI:** 10.3389/fmicb.2025.1611562

**Published:** 2025-07-02

**Authors:** Florian Reckinger, Anne-Marie Luther, Jutta Verspohl, Johann Lotz Artavia, Dagmar Waberski

**Affiliations:** ^1^Unit for Reproductive Medicine, Clinic for Swine and Small Ruminants, University of Veterinary Medicine Hannover, Hannover, Germany; ^2^Institute for Microbiology, University of Veterinary Medicine Hannover, Hannover, Germany; ^3^Mejoramiento Porcino, Barva, Costa Rica

**Keywords:** boar semen, climate change, resistant bacteria, semen transport, vibration emission, semen preservation

## Abstract

In a changing climate, the increase in temperature of boar semen doses during shipping to sow farms is a concern. Temperatures above the recommended storage range of 16–18°C for boar semen may compromise sperm quality due to bacterial growth and heat stress. The aim was to investigate whether cooling semen doses to 5°C prior to simulated transport at an environmental temperature of 30°C could inhibit bacterial growth and maintain sperm quality. Extended semen was treated in three variants: with and without pre-cooling to 5°C before simulated transport the next day at 30°C, and a control held stationary at 17°C. Transport vibration was simulated by shaking the semen doses for 6 h on an orbital shaker. Thereafter, all samples were stored at 17°C for 144 h. Pre-cooling efficiently delayed exponential microbial growth in samples spiked with resistant bacterial species. Although sperm motility was reduced by ~13 % in the pre-cooled samples, minimum quality requirements were fulfilled. Sperm membrane and mitochondrial membrane potential were not affected by the treatment. In conclusion, pre-cooling of semen before transport in a hot environment enhances biosafety of semen doses, while maintaining quality standards for use in artificial insemination.

## 1 Introduction

Global warming is an increasing concern for porcine fertility. In addition to the direct effects of heat stress on the reproductive performance of boar and sows (Knox, 2024), the exposure of spermatozoa to elevated temperatures during the long-distance transport of semen doses is becoming a growing concern. Typically, liquid preserved semen portions are shipped from boar studs to sow farms in insulated containers, such as Styrofoam boxes, equipped with gel-packs for passive cooling. Due to economic reasons, active cooling systems are not commonly used. Hence, in a changing climate and in tropical countries, maintaining the ideal storage temperature between 16 and 18°C during transport is challenging. Transport distances within or across countries may exceed 1,000 km and can last for 20 h or longer (Hafemeister et al., 2022; Wang et al., 2024), rendering an increase of semen temperature unavoidable. For example, semen doses transported for 27 h during a summer day in Nebraska, USA, arrived with a temperature of 25°C (Gonzalez-Castro et al., 2022). High arrival temperatures can lead to the rejection of semen portions by farmers due to concerns about heat-induced sperm deterioration and energy exhaustion, which can have a significant impact on fertility. Importantly, in addition to the direct heat stress effect on spermatozoa, high temperatures increase the risk for microbial growth, especially if resistant bacteria are present in the semen portion. Typically, boar semen contains mesophilic bacteria (Althouse and Lu, 2005; Costinar et al., 2021), which display growth optima between 20 and 40°C in a characteristic exponential course. Therefore, long-term shipping in a hot environment increases the risk of bacterial loads that are sperm-damaging or cause infections in the inseminated sows. Facing the climate change together and the observed increase of antimicrobial resistance in extended semen (Waberski et al., 2019b), solutions for biosafe semen shipping are urgently needed.

In addition to temperature fluctuations, vibration emission associated with shipping in cars and airplanes was identified as a risk for sperm quality (Schulze et al., 2018). Several *in vitro* studies have demonstrated that transport-simulated shaking can deteriorate sperm membrane integrity and inhibit motility, mitochondrial function (Schulze et al., 2018; Paschoal et al., 2021), as well as glycolysis and capacitation signaling (Wang et al., 2024). In agreement with these reports, moderate shaking of semen doses using an orbital shaker at 200 rpm for 3 h reduced *in vivo* fertility (Wang et al., 2024).

One potential way to mitigate semen warming during transport is to cool the semen doses to 5°C prior to shipping. This temperature was originally chosen to inhibit the growth of resistant bacteria, especially *Serratia marcescens* and *Klebsiella oxytoca* (Maassen et al., 2023). A higher temperature (e.g., 10°C) would be preferable for preventing chilling injury in boar spermatozoa; however, it does not sufficiently limit bacterial growth during long-term semen storage (Waberski and Luther, 2024).

Pre-cooling the semen could not only delay semen warming during transport but also render the semen doses less susceptible to vibration-induced mechanical stress (Paschoal et al., 2021; Hensel et al., 2024). Cold-semen preservation has recently been established for boar semen (Waberski et al., 2019a) and was since then approved by extensive *in vitro* studies and *in vivo* fertility trials (Jakel et al., 2021b; Reckinger et al., 2025). Still, for long-distance transport, especially in airplanes, shipping in actively refrigerated boxes is logistically and economically infeasible for the pig industry, and 5°C semen storage on farms is not yet established. We therefore propose an alternative solution: shipping semen pre-cooled to 5°C in commonly used Styrofoam boxes. We assume that during long-distance transport in a hot environment, the semen would gradually warm up, thereby limiting the temperature increase to levels that are tolerable for spermatozoa. We further hypothesize that the microbiological status and quality of semen in pre-cooled doses during subsequent semen storage at 17°C are improved compared to samples that were not pre-cooled.

Thus, the first aim was to establish a cooling curve in our laboratory that simulates a typical temperature profile encountered during long-distance semen transport, as reported from an AI center in a tropical country. Secondly, the transport effects of pre-cooled semen on the microbiological status and sperm functionality were compared to not pre-cooled semen, and to semen consistently stored unmoved at 17°C (control), thereby reflecting ideal storage conditions. To enhance the real-world simulation, we mimicked mechanical transport effects by exposing the semen portions to vibration emission in hot environmental temperatures.

## 2 Material and methods

### 2.1 Chemicals and media

Chemicals were purchased from Sigma-Aldrich Productions GmbH (Steinheim, Germany) and were of analytical grade. The semen extender Androstar^®^ Premium (APrem) containing an organic bactericide supplement and antibiotics was obtained from Minitüb GmbH (Tiefenbach, Germany). For microbiology, culture media with sheep blood produced by Thermo Fisher Scientific GmbH (Wesel, Germany) were used. For flow cytometric analysis, fluorescent dyes were purchased from Biozol Diagnostica GmbH (Hamburg, Germany), Thermo Fisher Scientific GmbH and Biomol GmbH (Hamburg).

### 2.2 Semen samples

Semen was collected via the gloved hand method from ten healthy, sexually matured boars located at the Unit for Reproductive medicine, University of Veterinary Medicine Hannover. The boars, aging between 1 and 6 years, were of Piétrain, Landrace, and Large White breeds. The animals were housed solitary straw-lined units, each at least 6 m^2^ in size, and cared for following the European Commission Directive for Pig Welfare following the ARRIVE guidelines. All animal-related procedures were approved by the Animal Welfare Committee of the University of Veterinary Medicine Hannover. Normospermic whole ejaculates, devoid of bulbourethral gland secretion, were extended with APrem containing 0.125 mg/mL Gentamicin sulfate to 20 × 10^6^ sperm/mL. The extended semen from each boar was categorized into three different groups: pre-cooled semen subjected to vibration emission at 30°C; not pre-cooled semen also exposed to vibration emission at 30°C; and semen stored without movement at 17°C as the control group. Through the split-sample design used in this study, as well as repeated measurements at different storage times, the effect of individual boar could be minimized.

### 2.3 Temperature management and vibrations set up

The transport conditions for routine shipping of semen in a tropical climate were first analyzed and later replicated in laboratory experiments at the University of Veterinary Medicine in Hannover, Germany. In practical terms, semen portions were produced at a boar stud located in Costa Rica and then transported by airplane the next day to a sow farm in 600 km away while enduring temperatures of 30 ± 2°C. Semen extended in APrem was shipped using two methods: the conventional method, with packaging of 17°C-semen tubes, and a modified method, with semen tubes pre-cooled to 5°C. For the conventional method, semen doses were held for 2 h at 20°C and then stored overnight in a climate cabinet set to 17°C. Owing to the arrival temperature (25–27°C) of the semen doses on farm, the modified method was introduced, which involved pre-cooling the semen to 5°C. Keeping in mind the high chilling sensitivity of boar spermatozoa, the semen was gently cooled following the temperature course established for 5°C semen storage, starting with 3 h at 25°C and then undergoing a three-step cooling process to reach 17, 10, and finally 5°C (Paschoal et al., 2020). For this, 200 semen doses were placed overnight in a refrigerator at 5°C. The following morning, batches of 100 semen tubes were packed in insulated Styrofoam boxes with an external cardboard cover. Isothermic bags filled with water at 5°C were placed at the top and bottom layers, with semen tubes between them, for extra insulation. These packages were then transported to the airport, flown for 2 h, and then delivered to the farm. The interval between semen collection and unpacking of the semen tubes on farm was ~48 h. The temperature changes in the semen package during transit and upon arrival at the farm were monitored with data logger. By the end, conventionally shipped semen (without pre-cooling) had a temperature between 25 and 27°C, while pre-cooled semen was received at 16–18°C. Semen was then stored on the farm at 17°C in the dark prior to use.

**Figure 1:**
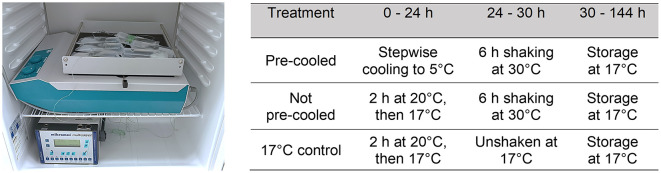
Temperature and vibration emission in the three semen groups. Pre-cooled and not pre-cooled semen doses were horizontally placed on an orbital shaker in a programmable climate cabinet that simulated the temperature course during transport in a hot environment. Temperature was recorded outside and inside the semen tubes with multiple-channel datalogger (Mikromec1 Multisens MLm 424, Technetics GmbH, Freiburg, Germany). Shaking was performed at 200 rpm for 6 h. Control samples remained unshaken at 17°C.

**Figure 2:**
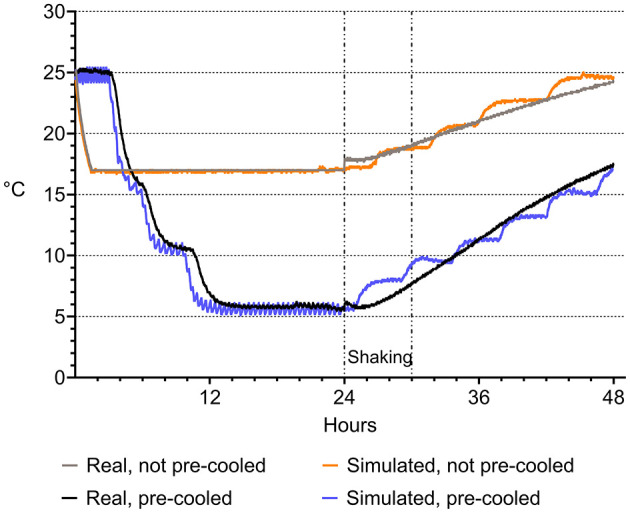
Temperature in semen doses during transportation in a hot environment. Temperature course was recorded during a real transport situation in a hot climate zone in two variants, with semen doses conventionally cooled to 17°C (“real not pre-cooled”) and semen doses pre-cooled to 5°C (“real pre-cooled”) before road and air transport for 6 h. The transport situation was simulated under laboratory conditions by exposing not pre-cooled and pre-cooled semen doses to vibration emission on a programmable climate cabinet. There was a high agreement between the real-world and the simulated transport scenarios.

The real-world scenario was replicated under laboratory conditions and adapted for experimental requirements. Temperature programs were established in two different programmable climate cabinets (Minitüb GmbH, Tiefenbach, Germany) to replicate the real-life temperatures recorded during semen shipping in the tropical environment described. Temperature programing was performed as described by Paschoal et al. (2020). Temperature was recorded in 1-min intervals during cooling using a multiple-channel datalogger (Mikromec1 Multisens MLm 424, Technetics GmbH, Freiburg, Germany) equipped with a flexible sensor positioned in the center of a tube filled with water. The vibration emission during transport was simulated by using orbital shakers (Swip Shaker, Bühler KL-2, Edmund Bühler GmbH, Bodelshausen, Germany) placed within the climate cabinets set to 30°C (Figure 1). As depicted in Figure 2, the simulated temperature curves were highly concordant with the real-life ones. Shaking of semen tubes began after 24 h, using a rotation speed (frequency) of 200 rpm and 1 cm of amplitude (Paschoal et al., 2021) for 6 h. The settings of frequency and orbital direction were adapted from transportation field test conditions (Schulze et al., 2018; Paschoal et al., 2021).

### 2.4 Bacterial counts and sample spiking

In Experiment 1, semen of six boars was used with their original bacterial content. Bacterial counts were determined in the raw semen from a 10-fold serial dilution prepared in PBS ranging from 10^−1^ to 10^−8^. A volume of 100 μL of each dilution was plated on Columbia agar with sheep blood (Thermo Fisher Scientific GmbH, Wesel, Germany) and incubated for 24 h at 37°C under aerobic conditions. Bacterial colonies were counted, and total bacterial numbers were calculated and expressed as colony-forming units per milliliter (CFU/mL). The bacterial species were identified with MALDI-TOF MS (microFlex LT, Bruker Daltonics GmbH & Co. KG, Bremen, Germany) with the software Biotyper (Bruker Daltonics, Server Version 4.1.100).

In Experiment 2, semen of six boars was spiked with gentamicin-resistant *K. oxytoca* (Experiment 2a) or gentamicin-resistant *S. marcescens* (Experiment 2b), both isolated from commercial semen portions and stored at −80°C before use. At 24 h prior to spiking, the bacterial isolates were cultured on sheep blood agar and incubated overnight at 37°C. Bacteria were diluted in 2 mL APrem extender and bacterial concentrations were adjusted after density photometry (SDM5, Minitüb GmbH, Tiefenbach, Germany). Extended semen samples were spiked with the bacterial solutions to a final concentration of ~4.5 × 10^3^ CFU/mL.

The bacterial counts in the semen subjected to the three different temperature/vibration treatments (see Section 2.2) were determined at 0, 24, 48, 72, and 144 h.

### 2.5 Sperm membrane integrity and functional parameters

In Experiment 3, extended semen from ten boars, each subjected to three different temperature/vibration treatments (see Section 2.2), was analyzed at 48 and 144 h.

Sperm kinematics were assessed using computer-assisted sperm analysis (CASA) and AndroVision^®^ Software (Version 1.2, Minitüb GmbH, Tiefenbach, Germany), as described previously (Maassen et al., 2023). All sperm cells exhibiting a velocity curved line (VCL) >24 μm/s and an amplitude of lateral head displacement (ALH) more than 1 μm, were identified as motile. Sperm demonstrating progressive motility were marked by a VCL higher than 41 μm/s and a velocity straight line (VSL) exceeding 15 μm/s. Additionally, parameters such as VCL (μm/s), (ALH; μm), linearity (LIN) and beat cross frequency (BCF; Hz) were recorded.

Flow cytometry was conducted following the protocols outlined in reference (Paschoal et al., 2020), using the Cyto Flex flow cytometer (Beckman Coulter GmbH, Krefeld, Germany) equipped with three lasers (488, 638, 405 nm). Gating was executed with CytExpert 2.4 Software (Beckman Coulter GmbH). Fluorescence signals from fluorescein isothiocyanate-conjugated peanut agglutinin (FITC-PNA), Yo-Pro-1 and Fluo3-AM were detected via a 525/40 nm band-pass filter. Hoechst 33342 signals were gated via a Pb 450/45 nm band-pass filter, propidium iodide (PI) signals via a PC 5.5 690/50 nm band-pass filter, and Merocyanin (M) 540 and JC-1 signals via a PE 585/42 nm band-pass filter. Sperm membrane integrity was evaluated using the stains PI and fluorescein FITC-PNA. Spermatozoa identified as negative for both PI and FITC-PNA were recorded as having intact membranes. Sperm membrane fluidity in viable sperm was assessed using M540 and Yo-Pro-1 stains. Spermatozoa negative for both Yo-Pro-1 and M540 were recorded as viable with low membrane fluidity. The mitochondrial membrane potential (MMP) in viable sperm was analyzed using the stains JC-1 and PI. Spermatozoa negative for PI and positive for JC-1 were recorded as viable sperm with high MMP. Hoechst 33342 stain was used to distinguish non-DNA particles, and the sperm population was gated based on forward- and side-scatter signals. Analysis was performed on 10,000 events per sample.

### 2.6 Statistical analysis

Data analysis was performed with SAS Studio (SAS Institute Inc., Cary, NC, USA, Enterprise 3.81). Spermatology data were checked for normal distribution and analyzed using one-way ANOVA individually for each examination time point. If the factor semen treatment was significant, a least square means comparison using Tukey's test was added. Additionally, the coefficient of determination (*R*^2^) was calculated to control the fitting of the model. Bacterial growth data were checked for normal distribution using the Kolgomorov-Smirnov test and Shapiro–Wilk test. In cases where the data were not distributed normally, the non-parametric Friedman test and *post-hoc* Wilcoxon test with Bonferroni correction were performed for each examination time individually. Bacterial counts in the spiked experiments varied widely over the long storage period, which was a decision factor in choosing a non-parametric analysis. To present these data, 95% confidence intervals were calculated. Power analyses were performed for both the microbiological and spermatological experiments. Spermatology data are presented as mean ± standard deviation, whereas microbiology data are shown as mean ± standard error of mean. All *p-*values < 0.05 were considered statistically significant.

## 3 Results

### 3.1 Bacterial counts

**Figure 3:**
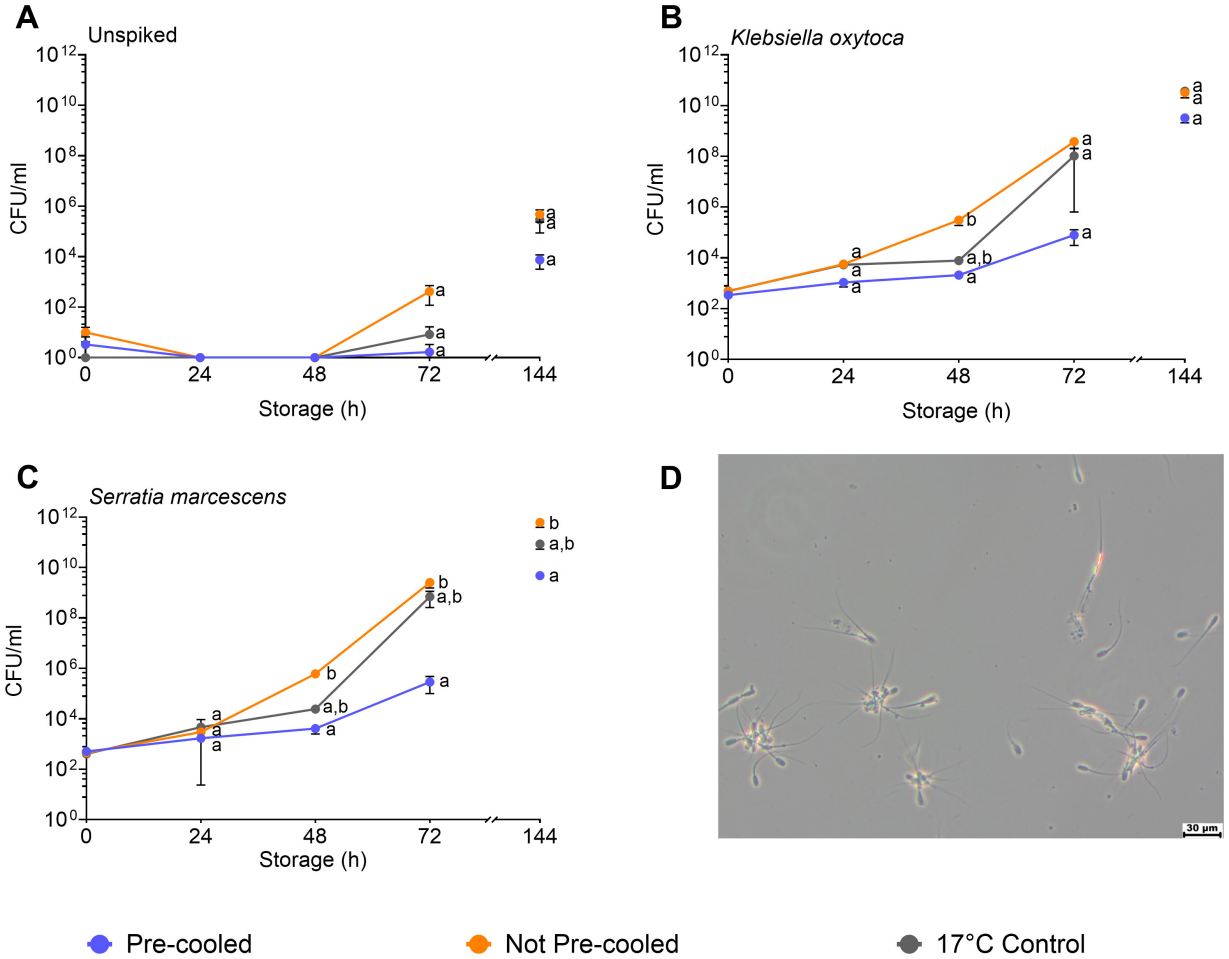
Effect of cooling of semen doses before simulated shipping in a hot environment on bacterial counts (CFU/mL) during transport (24–48 h) and during storage up to 144 h at 17°C. Semen doses remained unspiked **(A)** or were spiked with *Klebsiella oxytoca* **(B)** or *Serratia marcescens* **(C)**. Samples with *Serratia marcescens* showed a high degree of sperm agglutination at 72 h of storage **(D)**. Means and SEM of semen samples from six boars are shown. Significant differences between treatment groups within time points are marked with lower case letters (*p* < 0.05).

The raw semen contained between 1.7 × 10^3^ and 2.5 × 10^4^ CFU/mL (mean of 1.3 × 10^4^ CFU/mL), whereby gram-positive bacteria were identified in seven cases and gram-negative bacteria in five cases (Table 1). Semen extension reduced the bacterial counts by around one log level. After 72 h and 144 h storage, the highest bacterial counts (4.2 × 10^2^ and 4.8 × 10^5^) were found in the not pre-cooled semen samples. In the pre-cooled samples, the bacterial counts were 1.5 and 2 log levels lower compared to not pre-cooled semen (Figure 3A, Experiment 1). Semen spiked with *K. oxytoca* (Experiment 2a) reached bacterial counts of 3.8 × 10^8^ CFU/mL in the not pre-cooled samples, and 1 × 10^8^ CFU/mL in the 17°C control (Figure 3B). Bacterial counts in the pre-cooled semen samples were 2 log levels lower at 48 h (2.1 × 10^3^ CFU/mL), and 4 log levels lower at 72 h (7.9 × 10^4^ CFU/mL). Semen spiked with *S. marcescens* (Experiment 2b) revealed an exponential increase in not pre-cooled samples and in the 17°C-control, reaching log 9 levels of CFU/mL within 72 h of semen age. Pre-cooling reduced bacterial counts by 2 log levels at 48 h and by 4 log levels at 72 h compared to not pre-cooled semen (*p* < 0.05; Figure 3C). At 72 h, bacterial counts had reached 2.5 × 10^9^ CFU/mL, causing a high degree of sperm agglutination (Figure 3D). In both experiments with spiked samples, bacterial growth continued until 144 h to log levels greater than 10^8^ CFU/mL. In the spiked samples, the 95 % confidence limits varied around one log level within a treatment group (Supplementary Table 1). Power analysis of the spiked sample experiments showed high power (>0.99) for all groups spiked with *S. marcescens* and *K. oxytoca*, except the not pre-cooled group spiked with *S. marcescens* (0.22). This lower power can be attributed to the high variance in bacterial growth already after 48 h.

**Table 1:** Bacterial count (CFU/mL) and bacterial species in the raw semen (*n* = 6 boars).

**Bacterial count**	**Mean**	**SEM**	**Min**	**Max**
Bacterial count (CFU/ml)	1.3 × 10^4^	3.4 × 10^3^	1.7 × 10^3^	2.5 × 10^4^
**Bacterial species**				
Gram-negative	*Pseudomonas aeruginosa; Providencia stuartii; Escherichia coli; Klebsiella pneumoniae; Pasteurella aerogenes; Citrobacter koseri; Pantoea agglomerans*			
Gram-positive	*Staphylococcus* (coagulase-negative); *Corynebacterium; Enterococcus faecalis;* ß-hemolytic *Streptococcus*; *Bacillus* spp.			

### 3.2 Sperm membrane integrity and functional parameters

**Figure 4:**
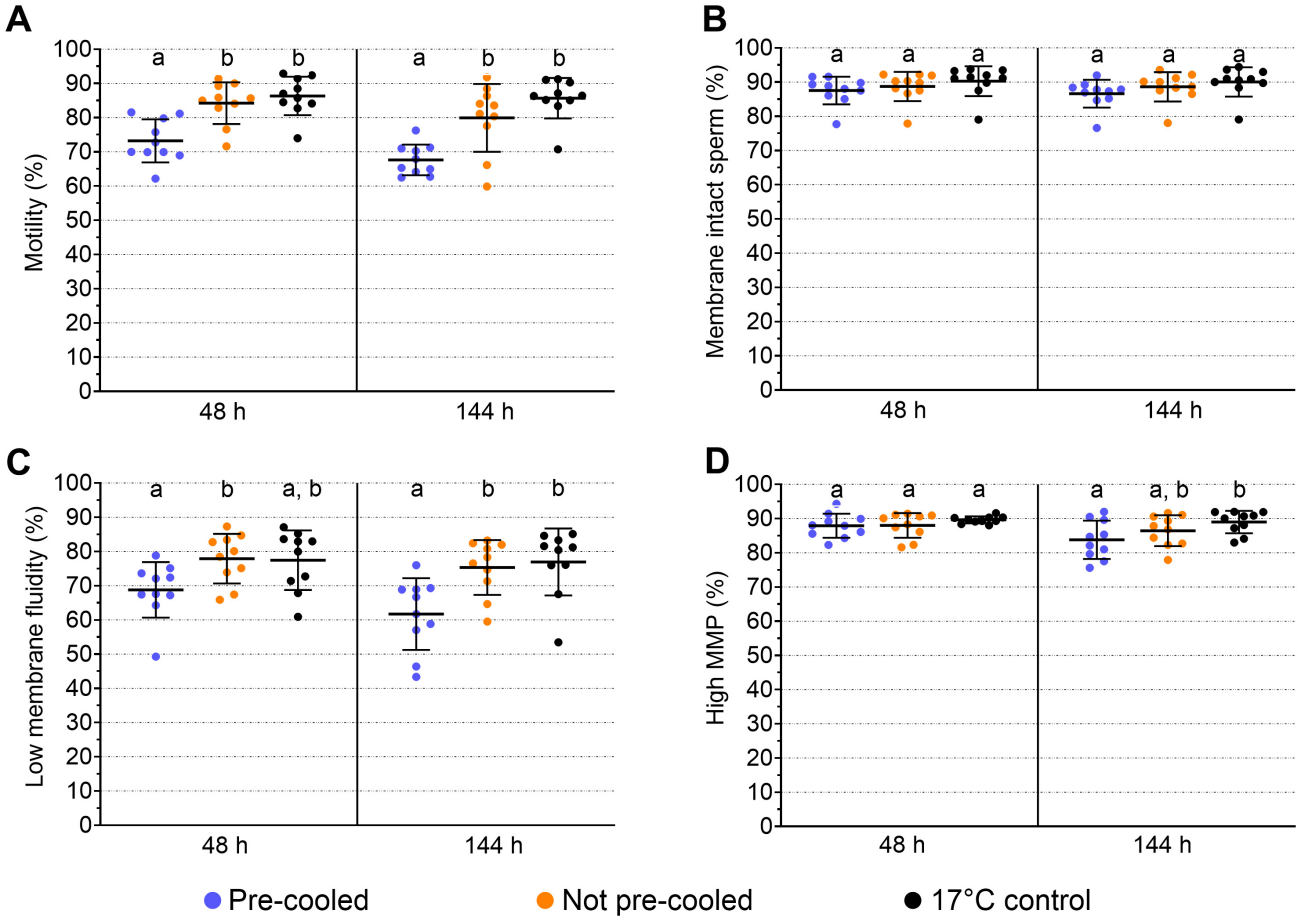
Effect of cooling of semen doses before simulated shipping in a hot environment on sperm quality at the end of transport (48 h) and after storage up to 144 h at 17°C. Means and SD of semen samples from ten boars are given for sperm motility **(A)**, membrane integrity **(B)**, viable sperm with low membrane fluidity **(C)**, viable sperm with high mitochondrial membrane potential (MMP; **D**). Significant differences between treatment groups within timepoints are marked with lower case letters (*p* < 0.05).

Spermatology data were evaluated after 48 h, pointing to the day of arrival on farm and the first usage of semen, and after 144 h for posing additional long-term storage stress on the spermatozoa (Experiment 3). Sperm motility differed among the semen groups at both time points (Figure 4A). The coefficient of determination in ANOVA for motility was *R*^2^ = 0.505. Thus, 50.5 % of the variance in motility values could be explained by the semen treatment. The power of the ANOVA motility model was >0.992. At 48 h and 144 h, average sperm motility was 73.2 ± 6.3 % and 67.6 ± 4.5 % in the pre-cooled semen, whereas in not pre-cooled semen 84.2 ± 6.1 % and 79.9 ± 9.9 % motile sperm were recorded. Sperm motility values did not differ between the not pre-cooled samples and the 17°C control (*p* > 0.05). Further sperm kinematics reveal lower values at 48 h for progressive motility, VCL and LIN, and at 144 h additionally for ALH in the pre-cooled compared to not pre-cooled samples (Table 2). There were no differences in sperm kinematics on either time point between the not pre-cooled and the 17°C control semen. Average percentages of spermatozoa with intact plasma membrane and acrosome varied between 86.6 ± 3.8 % and 90.3 ± 4.1 % and did not differ among the semen groups at both storage time points (*p* > 0.05; Figure 4B). The statistical power of the model for sperm membrane integrity was low, with values ranging between 0.2 and 0.27. No effect of sperm aging over the storage time was detected in either motility or functional parameters, which may be attributable to the use of a long-term semen extender. Pre-cooled semen showed a lower proportion of viable spermatozoa with low membrane stability (48 h: 68.8 ± 7.7 %; 144 h: 61.7 ± 9.9 %) compared to not pre-cooled samples (48 h: 77.9 ± 6.9 %; 144 h: 75.3 ± 7.6 %; Figure 4C). At 48 h and 144 h, membrane fluidity showed the greatest variability, with standard deviations of 8.1 and 10.5 % in the pre-cooled group, 7.2 % and 8.0 % in the not pre-cooled, and 9.8 % and 8.7 % in the control group, respectively. There was no difference (*p* > 0.05) between the pre-cooled and not pre-cooled groups in the percentage of viable sperm with high mitochondrial membrane potential (Figure 4D). The percentage of viable sperm with high mitochondrial membrane potential was above 75% at each timepoint.

**Table 2:** Sperm kinematics in three semen groups during storage at 17°C after different simulated transport conditions; *n* = 10 boars.

**Sperm parameters**	**Pre-cooled, shaked**		**Not pre-cooled, shaked**		**17°C control unshaked**	
	**Mean**	**SD**	**Mean**	**SD**	**Mean**	**SD**
**48 h**						
Prog. motility (%)	68.5^a^	8.1	80.3^b^	7.3	82.5^b^	8.1
Min–Max	56.9–80.4		67.7–89.0		63.9–92.1	
VCL (μm/s)	119.2^a^	19.0	146.9^b^	29.2	141.6^a, b^	20.4
Min–Max	78.5–145.7		114.1–190.1		108.4–172.2	
BCF (Hz)	24.3	2.0	23.9	2.5	25.9	3.3
Min–Max	22.0–27.7		20.4–28.1		22.3–32.4	
ALH (μm)	0.96	0.17	1.12	0.20	1.10	0.17
Min–Max	0.67–1.25		0.87–1.41		0.85–1.41	
LIN	0.44^a^	0.06	0.51^b^	0.06	0.49^a, b^	0.06
Min–Max	0.34–0.51		0.41–0.59		0.40–0.57	
**144 h**						
Prog. motility (%)	61.1^a^	6.0	73.3^b^	14.1	81.2^b^	6.8
Min–Max	54.4–69.5		44.7–91.0		67.7–90.5	
VCL (μm/s)	99.7^a^	18.1	134.6^b^	38.0	152.0^b^	19.4
Min–Max	63.2–123.7		61.0–188.6		112.1–185.3	
BCF (Hz)	19.5^a^	2.5	21.7^a, b^	3.3	24.5^b^	3.6
Min–Max	14.4–22.7		16.0 – 27.0		18.5–31.1	
ALH (μm)	0.83^a^	0.15	1.07^b^	0.28	1.18^b^	0.15
Min–Max	0.56–1.05		0.55–1.59		0.99–1.53	
LIN	0.49	0.07	0.48	0.07	0.48	0.05
Min–Max	0.38–0.60		0.38–0.56		0.40–0.55	

Different superscript letters present significant differences in Tukey test within a given storage time (*p* < 0.05).

## 4 Discussion

Our study shows that pre-cooling of semen before lengthy and agitation-intense transport in a hot environment can prevent excessive temperatures that promote bacterial growth and also maintain sperm quality at a level suitable for use in insemination.

In the real-world scenarios, such as an AI center in a tropical country, the slow pre-cooling of 200 semen doses before long-distance shipping in Styrofoam boxes was realized overnight in accordance with a cooling curve specifically designed for 5°C semen storage (Paschoal et al., 2020). At an environmental temperature of 30°C, this resulted in a semen temperature at 17°C upon arrival on farm, which is the typical storage temperature for boar semen (Riesenbeck, 2011). In contrast, using the standard procedure of shipping uncooled semen resulted in warming of semen doses to 25°C. Based on this practical findings, conditions were replicated in our laboratory, including a 6-h phase of agitation to simulate vibrations experienced during road and air transport.

Besides the impairment of sperm quality, the potential risk for an increased bacterial growth in warmed semen presents a significant concern. Bacteria are a natural component of semen and their growth is normally suppressed by antibiotic additives to semen extenders. Our data suggest that, even if semen samples were warmed to 25°C during the 24 h transport, antimicrobial control remains effective. The tendency to higher bacterial counts in the uncooled semen is not relevant given that semen is typically used for insemination within 3 days, when the bacterial counts are still below threshold levels (10^6^ CFU/mL) for sperm damage (Althouse et al., 2000; Sepulveda et al., 2014; Prieto-Martinez et al., 2014; Luther et al., 2023b). However, the situation changes if the semen samples contain drug-resistant bacteria. In fact, the prevalence of multi-drug resistance in boar semen is increasing, attributable to intense use of antibiotics over many years (Schulze et al., 2020). Of the resistant bacteria detected in extended boar semen, *S. marcescens* and *K. oxytoca* pose the highest risk to sperm survival due to their rapid growth and high cell toxicity (Luther et al., 2023a). Using these bacteria as indicators for resistant microbes in spiked semen samples, the present study clearly demonstrates that pre-cooling semen to 5°C prior to transportation effectively reduces the growth rate of bacteria during post-shipping semen storage at 17°C. During the first 3 days (72 h), bacterial load in the pre-cooled samples was maintained at a low level that would not harm sperm quality. The threshold for the spermicidal effect of *S. marcescens* and *K. oxytoca* is around log 7 CFU/mL (Luther et al., 2023a,b). In the not pre-cooled samples, these bacterial counts were already exceeded after 72 h of storage, reaching levels of log 8–9 CFU/mL, which results in sperm agglutination and loss of motility. Thus, pre-cooling of semen for 12 h at 5°C prior to shipping is clearly advantageous, as it helps prevent excessive bacterial growth that could cause damage to sperm already within the first 3 days of semen age. As expected, rapid exponential growth of *S. marcescens* and *K. oxytoca* continued during long-term storage for more than 72 h at 17°C. This could be avoided by continuous storage of the semen doses at 5°C, as shown previously (Maassen et al., 2023).

Our recent *in vitro* studies comprising molecular markers for capacitation, sperm-oviduct binding, and DNA integrity have shown that the cold storage concept maintains essential sperm functionality (Waberski et al., 2019a; Jakel et al., 2021a). This was confirmed by a series of field fertility trials with more than 2,000 sows on different farms (Waberski and Luther, 2024). In those trials, following controlled cooling, semen was consistently stored at 5°C until use. This differs from the present experiment, where semen was re-warmed after cooling to reflect a real-world scenario in which active cooling during long distance transport is not feasible. Although pre-cooling slightly reduced sperm motility (in average 11 % at 48 h), the preserved semen still fulfilled the minimum standards required for artificial insemination (BRS, 2023). The cooling and subsequent rewarming process—specifically the 12°C temperature change from 5°C to 17°C—might impose greater stress on spermatozoa compared to conventionally processed semen, which only experiences an 8°C change during warming from 17 to 25°C. Additionally, the semen pre-cooled to 5°C experienced a shorter temperature adaptation period (12 h) than the semen kept at 17°C (24 h) before exposure to vibration stress. An extended holding time for 16–24 h at 17°C is beneficial for the quality of semen subsequently cooled and stored at 5°C (Luther et al., 2024). However, longer holding is impracticable for long-distance shipping due to ongoing semen aging. As also indicated by an enhanced membrane fluidity, the aforementioned stressors could cause the slight reduction in sperm quality. It is to note that the membrane integrity, often referred to as “viability”, and the mitochondrial membrane potential remained unaffected by pre-cooling, thus confirming previous observations regarding the high resistance of chilled sperm to agitation effects (Paschoal et al., 2021; Hensel et al., 2024). As a limitation of this study, additional molecular sperm markers (e.g., tyrosine phosphorylation, acrosome reaction) and epigenetic consequences (e.g., histone transitions, DNA methylation shifts) that have been reported to be associated with cooling stress (Chen et al., 2024), were not examined. However, based on our previous studies with semen stored at 5°C, as outlined above, major effects of cooling-induced alterations in the extended sperm traits on fertility and offspring health are not expected.

A further important finding from the present study is that sperm quality was not affected in vibration-exposed, warmed semen when compared with unmoved 17°C controls, considered as gold standard. In practice, farmers often complain when semen arrives with 25°C. However, from a spermatology perspective, the concerns are not justified. This was supported by previous observations demonstrating that sperm kinematics, viability and the energy status in semen stored at 25°C do not differ from 17°C storage, even after 120 h of exposure to the elevated temperature (Henning et al., 2022). The situation could dramatically shift if the semen temperature rises above 25°C, or if drug-resistant bacteria with spermicidal properties are present. Exposure of 17°C semen samples in insulated Styrofoam boxes to 37°C for 24 h, followed by 17°C storage results in an irreversible loss of sperm motility and viability and promotes sperm apoptosis (Li et al., 2023). Similarly, short-term heat exposure (0.5 to 1 h) to temperatures of 40–41°C alters motion characteristics and metabolic profiles of boar spermatozoa, particularly affecting lipids and organic acids (Pena et al., 2021; Sui et al., 2022). In this study, we did not test these extreme conditions. Instead, we combined mild heat stress with stressors resulting from transport and subsequent long-term semen storage to closely mimic realistic conditions of semen shipping in a hot environment.

Importantly, in this study the long-term extender Androstar^®^ Premium was used, which, unlike the short term extender BTS, is suitable for cold-semen storage at 5°C (Waberski et al., 2019a) and avoids significant pH increases during agitation (Paschoal et al., 2021; Hensel et al., 2024). Although the composition of the Androstar^®^ Premium extender is not disclosed by the manufacturer, it is known to contain membrane protective and capacitation inhibiting ingredients, which may mitigate transport-associated stressors. In any case, for long-distance semen transports where anticipated challenges include agitation stress and temperature fluctuations, using a powerful extender media is recommended.

In conclusion, by simulating real-world conditions of long-distance semen transport in a warm climate, we demonstrated that pre-cooling semen to 5°C delays the exponential phase of bacterial growth, thereby enhancing the biosafety of semen doses. Warming to 25°C does not adversely affect sperm quality, provided that resistant bacteria known for their high sperm toxicity are absent. Controlled semen cooling in a protective extender prior to shipments prevents heat-induced alterations in the microbiological status and will increase the farmer's confidence in the delivered product.

## Data Availability

The raw data supporting the conclusions of this article will be made available by the authors, without undue reservation.
