# Supplementary material for: Pre-cooling of boar semen before transport in a hot environment enhances biosafety
Source: Front Microbiol. 2025 Jul 2;16:1611562. doi: 10.3389/fmicb.2025.1611562 (PMC12263611; doi:10.3389/fmicb.2025.1611562)
Supplement: Supplementary file 1 [file Table_1.docx]

Tab. 1: Means and 95 % confidence limits (CL) of bacterial counts in Experiments 1 and 2 for each examination time. A) Samples spiked with *Klebsiella oxytoca*; B) Samples spiked with *Serratia marcescens*

A) Samples spiked with *Klebsiella oxytoca*

| **Klebsiella oxytoca** | pre-cooled | not pre-cooled | control |
| --- | --- | --- | --- |
|  |  | 0h |  |
| Mean | 3.4E+02 | 4.83E+02 | 4.93E+02 |
| lower 95% CL | 1.6E+02 | 2.11E+02 | 2.82E+02 |
| upper 95% CL | 5.2E+02 | 7.56E+02 | 7.05E+02 |
|  |  | 24h |  |
| Mean | 1.1E+03 | 5.67E+03 | 5.35E+03 |
| lower 95% CL | 1.6E+02 | 2.96E+03 | 1.69E+03 |
| upper 95% CL | 2.0E+03 | 8.38E+03 | 9.01E+03 |
|  |  | 48h |  |
| Mean | 2.1E+03 | 3.06E+05 | 7.78E+03 |
| lower 95% CL | 1.02E+03 | 3.64E+03 | 4.43E+03 |
| upper 95% CL | 3.18E+03 | 6.07E+05 | 1.11E+04 |
|  |  | 72h |  |
| Mean | 8.0E+04 | 3.79E+08 | 1.04E+08 |
| lower 95% CL | -4.5E+04 | -7.90E+07 | -1.61E+08 |
| upper 95% CL | 2.0E+05 | 8.36E+08 | 3.70E+08 |
|  |  | 144h |  |
| Mean | 3.2E+09 | 3.21E+10 | 3.68E+10 |
| lower 95% CL | 2.7E+08 | 7.40E+09 | -5.92E+09 |
| upper 95% CL | 6.2E+09 | 5.68E+10 | 7.95E+10 |

B) Samples spiked with *Serratia marcescens*

| **Serratia marcescens** | pre-cooled | not pre-cooled | control |
| --- | --- | --- | --- |
|  | 0h | | |
| Mean | 5.1E+02 | 4.50E+02 | 4.07E+02 |
| lower 95% CL | -2.6E+01 | 8.91E+01 | 2.64E+01 |
| upper 95% CL | 1.0E+03 | 8.11E+02 | 7.87E+02 |
|  |  | 24h |  |
| Mean | 1.7E+03 | 2.98E+03 | 4.67E+03 |
| lower 95% CL | 8.5E+02 | 4.82E+02 | 4.34E+02 |
| upper 95% CL | 2.6E+03 | 5.48E+03 | 8.90E+03 |
|  |  | 48h |  |
| Mean | 4.1E+03 | 6.03E+05 | 2.43E+04 |
| lower 95% CL | -5.00E+00 | 2.84E+05 | 1.20E+04 |
| upper 95% CL | 8.21E+03 | 9.21E+05 | 3.66E+04 |
|  |  | 72h |  |
| Mean | 2.9E+05 | 2.54E+09 | 7.00E+08 |
| lower 95% CL | -2.0E+05 | -3.55E+07 | -4.37E+08 |
| upper 95% CL | 7.7E+05 | 5.11E+09 | 1.84E+09 |
|  |  | 144h |  |
| Mean | 4.8E+09 | 6.18E+11 | 8.54E+10 |
| lower 95% CL | 2.1E+09 | 4.49E+10 | 3.51E+09 |
| upper 95% CL | 7.5E+09 | 1.19E+12 | 1.67E+11 |
